# Is There a Relationship Between First-Trimester Aneuploidy Screening Serum Biomarker Values and Nuchal Translucency Measurements and the Development of Retinopathy of Prematurity (ROP) in Premature Infants?

**DOI:** 10.7759/cureus.46969

**Published:** 2023-10-13

**Authors:** Mehmet Fatih Küçük, Hasan Basri Savaş, Elcin Suren, Muhammet Erol, Lütfiye Yaprak, Senol Sabanci

**Affiliations:** 1 Ophthalmology, Health Sciences University, Antalya Training and Research Hospital, Antalya, TUR; 2 Medical Biochemistry, Faculty of Medicine, Mardin Artuklu University, Mardin, TUR; 3 Ophthalmology, Antalya Education and Research Hospital, Antalya, TUR

**Keywords:** first-trimester aneuploidy screening, nuchal translucency, pregnancy-associated plasma protein a, human chorionic gonadotropin, retinopathy of prematurity

## Abstract

Purpose

The purpose of this study is the evaluation of serum biomarker and nuchal translucency (NT) values measured during first-trimester aneuploidy screening in terms of the development of retinopathy of prematurity (ROP) in premature infants and investigation of whether the development of ROP is associated with these parameters.

Methods

In this retrospective cohort study, 3,750 premature infants who underwent ROP screening from 2016 to 2021 were identified from the hospital medical record system. Among 2,130 premature babies screened for first-trimester aneuploidy, 166 babies whose mothers had single pregnancies were screened by the same method and showed the same clinical course in both eyes were included in the study. The infants were divided into two groups according to the presence of ROP, and those with ROP were further evaluated in two groups according to the presence of proliferation. The groups were compared in terms of the serum values of human chorionic gonadotropin and pregnancy-associated plasma protein A, among aneuploidy screening biomarkers, and NT measurements.

Results

There was no significant difference in the evaluated serum biomarker values and NT measurements between the ROP and non-ROP groups or between the proliferative ROP, non-proliferative ROP, and non-ROP groups.

Conclusion

Our results showed that first-trimester aneuploidy screening serum biomarker values and NT measurements were not associated with the development of ROP in premature infants.

## Introduction

Retinopathy of prematurity (ROP) is a potentially blinding but preventable disease of retinal vascular structures in premature infants. The most important criteria used in the preventive screening examination are birth weight (less than 1,501 g) and gestational age (30 weeks and below) [1]. In addition, the development of ROP is associated with many risks, especially the duration of oxygen administration. Ongoing research has shown that there are many maternal serum biomarkers associated with these risks, and although many serum biomarkers are still under investigation, none has been shown to have 100% sensitivity and specificity [2]. There are articles in which the relationship between the values of biomarkers obtained from serum in aneuploidy screening and ROP risk factors, individual or in combination, is statistically analyzed. In some of these studies, low birth weight (LBW) and small for gestational age (SGA), which are considered to be the strongest risks, have been reported to be associated with maternal serum biomarkers, such as human chorionic gonadotropin (hCG) and pregnancy-associated plasma protein A (PAPP-A), while others detected no such association [3-5]. hCG is a pregnancy hormone produced by trophoblasts after implantation and stimulates the secretion of steroids necessary for the proper development of the embryo. PAPP-A is a protease that acts on insulin-like growth factor-binding proteins (IGFBPs). The measurement of nuchal translucency (NT) is also undertaken during first-trimester aneuploidy screening. NT refers to the collection of subcutaneous fluid behind the fetal neck, and a measurement of more than 3 mm is considered pathological. There are studies suggesting that NT values are also associated with SGA and LBW, which are important ROP risk factors [6], as well as authors suggesting that they are unrelated [7].

While there are many studies showing that the above-mentioned biomarkers are associated with ROP risk factors, only few studies have directly examined the association of these biomarkers with ROP [8]. While the relationship between maternal serum hCG and PAPP-A and ROP has been previously investigated, to the best of our knowledge, no study has been undertaken to perform NT measurements measured using ultrasound during aneuploidy screening. Therefore, in this study, we aimed to investigate the relationship between the development of ROP in premature infants and biomarker values measured from the serum samples and the NT values measured by ultrasonography during first-trimester aneuploidy screening.

## Materials and methods

The study was planned as a retrospective cohort study. After receiving approval from the local ethics committee (approval no: 2020/315), a total of 3,750 premature infants and their mothers, who presented to the hospital from 2016 to 2021, were identified, and their demographic characteristics, clinical findings, laboratory test results, and treatments were questioned through the medical records of our hospital. This inquiry was conducted following regulations regarding medical record management and confidentiality principles. All infants who met the ROP screening criteria defined in the American Academy of Pediatrics 2013 (birth weight ≤ 1,500 g or gestational age: 30 weeks or less) were included in ROP screening. In addition, infants with a birth weight of 1,500 to 2,000 g or a gestational age of >30 weeks but considered by neonatologists to have an unstable clinical course and high risk for ROP were also included in this examination [9]. Indirect ophthalmoscopy and cameras (RetCam Shuttle imaging device, Clarity Medical Systems Inc., Pleasanton, CA, USA and PedCam, a pediatric camera with a 200° field of view) were used in the examination of these premature infants, and wide-angle images were recorded. In premature infants with ROP (734 premature infants), the disease was classified according to the revised International Committee for the Classification of Retinopathy of Prematurity [10], and the treatment protocol was guided by the recommendations set forth by Early Treatment for Retinopathy of Prematurity Cooperative Group [11]. ROP follow-up and treatment were performed by three pediatric retina specialists (M.K.E., E.S., and M.F.K.). In patients who required treatment, injections and the use of diode lasers were performed after obtaining informed consent from their parents. Among the 3,750 premature infants, aneuploidy screening of the mother had been performed in 2,130 according to Committee Opinion 296 published by the American College of Obstetricians and Gynecologists in 2004 [12]. Hormone levels were measured with the electrochemiluminescence (ECL) method using the Roche Cobas e 601 (Roche Diagnostics, Indianapolis, USA) device with a Roche commercial kit.

Eligibility criteria

Premature infants with the same clinical findings in both eyes were included in the study, and the findings of only one eye (left) were used in statistical analysis. Multiple pregnancies (twins, triplets, and above) were excluded from the study. The results of laboratory tests that gave results in different units for PAPP-A and hCG were also excluded. Only premature infants whose measurements were made with the Roche Cobas e 601 device were included in the study.

Due to these strict exclusion criteria, only 166 premature newborns with first-trimester aneuploidy screening data were identified. The parents of the premature infants were informed about the study, and their written consent was obtained. Then, the infants were divided into two groups according to the presence of ROP. In the ROP group, 53 infants had ROP in both eyes, and the clinical stage in the left eye was the same as in the right eye in all control examinations. The non-ROP group consisted of 113 premature infants due to strict inclusion criteria. Then, the premature infants in the ROP group were further evaluated in the two groups as those with proliferative ROP (P-ROP) (24 premature), who recovered with treatment, and those with non-proliferative ROP (NP-ROP) (29 premature), who recovered without treatment. The clinical stage in both eyes of the premature infants in the P-ROP group was the same, and both eyes were treated equally with the same treatment method.

Descriptive characteristics, such as age and gestational age, were analyzed separately for the mothers and premature infants according to the presence of ROP. As biochemical markers of first-trimester aneuploidy screening, total hCG, free β-hCG, and PAPP-A, and their multiple of median (MoM) values, as well as NT measurements, obtained using ultrasound during the same screening and their MoM values were compared between the groups.

Statistical analysis

IBM SPSS Statistics for Windows, Version 22 (Released 2013; IBM Corp., Armonk, New York, United States) was used for statistical analyses. As descriptive statistics, frequency, percentage, mean (mean), standard deviation and median (median), and minimum and maximum values were used. The Shapiro-Wilk and Kolmogorov-Smirnov tests were conducted to evaluate whether the data conformed to a normal distribution (p > 0.05). The Mann-Whitney U test was used as one-way analysis of variance in the comparison of non-normally distributed variables between two groups, while the Kruskal-Wallis test was conducted as one-way analysis of variance in the comparison between three groups (post hoc test; Dunn’s). P values less than 0.05 were considered statistically significant.

## Results

In the study, a total of 90 female and 76 male premature infants were examined. There were 113 premature infants in the non-ROP group and 53 in the ROP group. Of the infants with ROP, 24 were in the P-ROP group and 29 in the NP-ROP group. The demographic characteristics and systemic clinical findings of the mothers of these infants are shown in Table 1. First-trimester aneuploidy screening was performed at an average of 86.37 + 5.174 (73-99) gestational days, and the average maternal weight was 63.7799 + 12.204 (40-107) kg at the time of screening. The demographic characteristics and systemic clinical findings of the premature infants are shown in Table 2. In addition to the findings in Table 2, one premature infant had aggressive posterior ROP but was not detected to have Down syndrome risk during screening. In the ROP group, an anti-vascular endothelial growth factor (VEGF) injection was applied to 20 of the premature infants, and a diode laser was applied to two, and both injection and laser were applied to a further two patients. A single dose was sufficient for the premature infants who received injections. There was no risk of Down or Edwards/Patau syndrome in the screening of any of the premature infants who underwent laser treatment. While ROP was not seen in eight of the 11 premature infants with a risk of Down syndrome, ROP developed in three, of whom two were treated with an anti-VEGF injection when they progressed to P-ROP. ROP did not develop in one premature infant with a risk of Edwards /Patau syndrome. Table 3 shows the comparison of the aneuploidy screening serum biomarker values and NT measurements according to the presence of ROP in premature infants, and Table 4 presents the comparison of the aneuploidy screening serum biomarker values and NT measurements between the P-ROP, NP-ROP, and non-ROP groups.

**Table 1:** Demographic characteristics and systemic clinical findings of the mothers according to the presence of ROP in their babies ROP: Retinopathy of prematurity; SD: standard deviation

	ROP (53 mothers of premature infants)	Non-ROP (113 mothers of premature infants)
Maternal age at birth (years) mean ± SD (range)	30.88 ± 5.74 (20-45)	29.73 ± 5.63 (19-43)
Use of assisted reproductive technology n (%)	5 (9.40)	3 (2.65)
Preeclampsia during pregnancy n (%)	6 (11.32)	12 (10.61)
Eclampsia during pregnancy n (%)	0	3 (2.65)
Gestational diabetes mellitus n (%)	7 (13.20)	10 (8.84)
Systemic disease during pregnancy n (%)	9 (16.98)	22 (19.46)
Fever and rash during pregnancy n (%)	1 (1.80)	3 (2.65)
Medication use during pregnancy n (%)	9 (16.98)	16 (14.15)
Exposure to X-rays during pregnancy n (%)	0	1 (0.88)
Smoking and alcohol usage during pregnancy n (%)	8 (15.09)	21 (18.58)

**Table 2:** Demographic characteristics and systemic clinical findings of premature infants according to the presence of ROP ROP: Retinopathy of prematurity; SD: standard deviation

			ROP (53 premature infants)	Non-ROP (113 premature infants)
Gender n (%)	Female		31 (58.49)	59 (52.21)
	Male		22 (41.50)	54 (47.78)
Gestational age at birth (week) mean ± SD (range)			29.73 ± 3.12 (24-36)	32.91 ± 2.05 (28-39)
Birth weight (gr) mean ± SD (range)			1,386.76 ± 456.01 (700-2,400)	1,972.54 ± 449.74 (950-3,930)
Type of childbirth n (%)		Vaginal delivery/natural childbirth	47 (88.67)	106 (93.80)
		Cesarean section	6 (11.32)	6 (5.30)
		Assisted vaginal delivery (breech birth)	0	1 (0.88)
Intubation n (%)			34 (64.15)	48 (42.47)
Sepsis n (%)			25 (47.16)	51 (45.13)
Necrotizing enterocolitis n (%)			4 (7.54)	4 (3.53)
Intracranial hemorrhage n (%)			2 (3.77)	2 (1.76)

**Table 3:** Comparison of aneuploidy screening serum biomarker values and NT measurements according to the presence of ROP in premature infants NT: Nuchal translucency; ROP: retinopathy of prematurity; hCG: human chorionic gonadotropin; MoM: multiple of the median; PAPP-A: pregnancy-associated plasma protein-A * Mann-Whitney U test

	ROP (53 premature infants)				Non-ROP (113 premature infants)				P*
	N	median	Min	Max	N	median	min	max	
Total hCG (IU/L)	53	123,940	45,900	370,869	113	89,224.50	43,000	297,700	0.542
Free β-hCG (IU/ml)	53	36.10	11.03	379.31	113	37.14	6.98	183.43	0.934
Total hCG MoM	53	1.28	0.43	3.03	113	1.17	0.57	4.23	0.591
Free β-hCG MoM	53	0.90	0.48	4.82	113	0.97	0.22	3.33	0.643
PAPP-A (ng/ml)	53	1,150.80	109.32	3,781.20	113	1,011.06	127.40	13,109	0.576
PAPP-A MoM	53	1.00	0.24	2.47	113	0.91	0.17	3.43	0.245
NT (mm)	53	1.52	0.90	2.50	113	1.30	0.60	2.20	0.093
NT MoM	53	0.99	0.52	1.51	113	0.86	0.49	2.10	0.128

**Table 4:** Comparison of aneuploidy screening serum biomarker values and NT measurements between the P-ROP, NP-ROP, and non-ROP groups NT: Nuchal translucency; P-ROP: proliferative retinopathy of prematurity; NP-ROP: non-proliferative retinopathy of prematurity; hCG: human chorionic gonadotropin; MoM: multiple of the median; PAPP-A: pregnancy-associated plasma protein-A Bold values represent statistical significance (P < 0.05); P, between the three groups; P1, between the P-ROP and NP-ROP groups; P2, between the P-ROP and non-ROP groups; P3, between the NP-ROP and non-ROP groups *Kruskal-Wallis test; ^§^Dunn’s test

	P-ROP (24 premature infants)				NP-ROP (29 premature infants)				Non-ROP (113 premature infants)				P^*^	P_1_^§^	P_2_^§^	P_3_^§^
	N	Median	Min	Max	N	Median	Min	Max	n	Median	Min	Max				
Total hCG (IU/L)	24	105,972	80,581	133,244	29	171,880	45,900	370,869	113	89,224.50	43,000	297,700	0.826	0.995	0.947	0.846
Free β-hCG (IU/ml)	24	28.13	17.84	379.31	29	45.48	11.03	379.31	113	37.13	6.98	183.43	0.250	0.222	0.495	0.621
Total hCG MoM	24	1.30	0.85	1.54	29	1.25	0.57	4.23	113	1.17	0.43	3.03	0.862	0.995	0.910	0.922
Free β-hCG MoM	24	0.88	1.31	0.50	29	1.05	1.65	0.48	113	0.97	1.67	0.22	0.686	0.747	0.993	0.701
PAPP-A (ng/ml)	24	1,016.89	258.93	3,781.20	29	1,284.80	109.32	2,794.80	113	1,011.06	127.40	13,109	0.716	0.824	1.000	0.703
PAPP-A MoM	24	0.95	0.24	2.29	29	1.05	0.26	2.47	113	0.91	0.17	3.43	0.507	0.995	0.706	0.586
NT (mm)	24	1.60	1	1.86	29	1.50	0.9	2.50	113	1.30	0.60	2.20	0.209	0.843	0.260	0.539
NT MoM	24	1	0.52	1.51	29	0.91	0.53	1.43	113	0.86	0.49	2.10	0.287	0.906	0.334	0.605

## Discussion

In this study, we found that the hCG and PAPP-A values, which are serum biomarkers used in first-trimester aneuploidy screening, did not significantly differ between the premature infants with ROP and controls without ROP. We also found no significant difference between the P-ROP, NP-ROP, and non-ROP groups. There was also no significant difference in the NT measurements between these three groups. To our knowledge, this study is important since it is the first to compare the first-trimester aneuploidy screening serum biomarkers and NT between premature infants with and without ROP. Our results indicate no relationship between ROP and maternal serum biomarker values and NT measurements, which have been previously shown to be associated with ROP risk factors in many studies.

hCG

When the free β-hCG, free β hCG MoM, total hCG, and total hCG MoM values measured during first-trimester aneuploidy screening were analyzed separately, it was found that there was no significant difference between the premature infants with and without ROP in any of these parameters. Moreover, these parameters also did not significantly differ between the P-ROP, NP-ROP, and non-ROP groups. In the literature, there are studies reporting a relationship between hCG and SGA, which is one of the most important ROP risk factors [3], as well as others suggesting no such relationship [4]. Contradictory results have also been published concerning the relationship between hCG and LBW, which is another important ROP risk factor [4,13]. In addition, some authors examining the relationship between risk factors and the cut-off points of the MoM values of these serum biomarkers have reported different results.

The hCG molecule was previously demonstrated in nine fetal tissues, including those in the eye, while the CG/LH receptor was found in many fetal organs, including the eye. The comparison of fetal and maternal blood hCG levels revealed much lower values in the former than in the latter [14]. While systemic hCG reaches the posterior segment of the eye by crossing the blood-retina barrier, the retina also synthesizes its own hCG [15]. It has been shown that the potent proangiogenic hormones hCG and LH [15], together with retinal ischemia resulting from low oxygen tension and high oxygen tension, are involved in VEGF regulation and angiogenesis in the human retina [16]. HCG and LH facilitate this regulation through the CG/LH receptors in the retina [15]. Studies have shown that as the stimulation of these receptors increases, retinal angiogenesis is promoted. However, only animal eye experiments have shown that VEGF levels decrease in the absence of CG/LH receptors, which are the receptors of these proangiogenic hormones, even under normoxic conditions [17-19]. The tight regulation of VEGF within the eye is critical for retinal angiogenesis during ocular maturation and subsequent years [20]. Sufficient levels of VEGF are required for regular retinal angiogenesis in premature infants after birth [20,21]. Since deficiency in VEGF levels causes avascular areas to remain in the retina, these avascularized areas over-stimulate VEGF expression by causing ocular ischemia, eventually leading to the development of retinal neovascularization [16,22]. To date, the relationship between human retinal angiogenesis and hCG has only been claimed in one study [8]. The authors found a relationship between low hCG and failure to develop angiogenesis, but they were not able to demonstrate a similar association between high hCG and development of angiogenesis (neovascularization). Nevertheless, they noted that hCG values were high in infants with P-ROP [8]. In light of these literature findings, we consider that the hCG levels not being related to the development of ROP in our study may have been because VEGF could not be produced to provide sufficient angiogenesis due to the early deprivation of placental hCG in premature infants. In other words, we think that hCG levels in the aneuploidy screening are not related to the development of ROP since hCG levels are measured from samples taken from the maternal blood in the first trimester before separation from the hCG producer placenta when there are sufficient hCG levels to balance VEGF production. This idea is supported by Movsas et al., who argued that hCG was of fetal origin rather than of placental origin in fetal blood and showed a low level of hCG at the fourth week when ROP appeared [8].

PAPP-A

When we compared the PAPP-A values and PAPP-A MoM values according to the presence of ROP in premature infants, we did not find any significant difference between the two groups. Even when we compared the P-ROP, NP-ROP, and non-ROP groups, there was still no significant difference. In other words, we did not find any change in the values of these serum biomarkers, even in patients with ROP requiring treatment, compared to the remaining premature infants. PAPP-A, a metalloproteinase, is secreted from many reproductive and non-reproductive organs, as well as syncytial trophoblasts, and it is also produced by many cells, such as fibroblasts, endothelial cells, and smooth muscle cells. PAPP-A cleavages IGFBP 2, 4, and 5 proteolytically [23,24]. All the IGFBPs on the cell surface have a greater affinity for insulin-like growth factor (IGF) than the IGF receptor has for IGF [25]. IGF released by the proteolytic effect of PAPP-A binds to the IGF receptor located near IGFBP on the cell surface and takes part in the control of cellular processes in embryo development, such as mitosis, cell migration, differentiation, and apoptosis [26]. The majority of IGFs (IGFI-II) are not free in fetal serum and are bound to six separate IGFBPs. In an animal experiment, it was shown that with the loss of maternal IGF-I in premature birth, ROP risk factors further reduced fetal IGF-I, and there was no normal retinal vascular development despite VEGF in the medium. In the same study, it was reported that IGF-I increased slightly with developing retinal hypoxia, which, in turn, increased retinal angiogenesis by elevating VEGF level [27]. In another study, it was demonstrated that IGF-1 was associated with the development of ROP in the serum of premature infants after birth [16]. However, it is also known that rhIGF-I/IGFBP3 infusion cannot prevent ROP in extremely premature infants [28] because poor oxygen control, which is one of the important risk factors for ROP, may be a factor affecting the strength of IGF-I. While the relationship between VEGF and hCG in NP-ROP and P-ROP has been demonstrated, the relationship between VEGF and PAPP-A, another aneuploidy screening serum biomarker, has not been clarified in the literature. Similarly, while many studies have shown PAPP-A to be associated with ROP risk factors, such as LBW [4,29] and SGA [5], there are also studies that found no association between PAPP-A and SGA [5]. Thus, in the literature, there is a disagreement concerning the relationship PAPP-A and ROP risk factors. In the current study, which is the first to examine whether the molecules involved in the development of ROP were associated with PAPP-A, the results did not indicate any relationship.

NT

Similar to the results in the first-trimester aneuploidy serum biomarkers, we did not find any significant difference between the ROP and non-ROP groups in terms of the NT MoM measurements. We determined that the NT MoM values of the premature infants with P-ROP also did not significantly differ from those without ROP or those with NP-ROP. There are theories that NT areas develop due to underdeveloped lymphatic drainage, altered composition of the extracellular matrix, and abnormalities in the heart and great arteries [30]. While the relationship between ROP risk factors and NT measurements has been examined in many studies, to our knowledge, there is no study examining the relationship between ROP and NT measurements. While there is research showing a relationship between NT measurements and SGA and LBW, which are important ROP risk factors [6], there are also those that did not find evidence of such a relationship [7]. In the current study, NT measurements did not differ according to the presence or proliferative nature of ROP.

Many studies have shown that SGA, LBW, and prolonged oxygen therapy are the leading risk factors for ROP. Some have also reported a relationship between LBW and SGA and aneuploidy screening serum biomarkers hCG and PAPP-A and NT, while others have claimed that these parameters are not unrelated. In light of these findings, in this study, we examined the relationship between serum biomarkers that are claimed to be associated with ROP risk factors and ROP and found no change according to the presence of ROP in premature infants, similar to one group of studies in the literature. An important characteristic of our study is that all the analyses were in premature infants, and we did not include healthy newborns as the control group. Given that previous studies examining the relationship between serum biomarker values and risk factors did not perform this analysis according to the presence of ROP or the presence of proliferation in ROP, our study is considered to be important. However, the hCG and PAPP-A values not being examined in fetal blood or in the period before P-ROP developed can be considered a limitation of our study.

## Conclusions

In the literature, there are conflicting findings concerning whether HCG and PAPP-A, which are first-trimester screening serum biomarkers, and nuchal translucency measurements are associated with the most important ROP risk factors, i.e., LBW and SGA. Our results revealed no significant difference in the first-trimester aneuploidy screening serum biomarker values and NT measurements between the premature infants with and without ROP. Additionally, our results revealed that there was no difference in the same biomarkers between the ROP cases requiring treatment, those that recovered without treatment, and those without ROP. Therefore, we suggest that first-trimester aneuploidy screening serum biomarker values and NT measurements are not associated with the development of ROP in premature infants.
